# Common food preservatives induce an oxidative stress response in Salmonella enterica serovar Typhimurium

**DOI:** 10.1099/mic.0.001609

**Published:** 2025-09-15

**Authors:** Emma R. Holden, Joshua C.I. Horton, Mark A. Webber

**Affiliations:** 1 Quadram Institute Bioscience, Norwich Research Park, Norwich, Norfolk, NR4 7UQ, UK; 2 Centre for Microbial Interactions, Norwich Research Park, Norwich, Norfolk, NR4 7UG, UK; 3 Norwich Medical School, University of East Anglia, Norwich Research Park, Norwich, Norfolk, NR4 7TJ, UK

**Keywords:** functional genomics, TraDIS, transposon mutagenesis

## Abstract

Despite their frequent use, the mechanisms of action of common food preservatives are poorly understood. As there is a drive to develop alternative preservatives, understanding the mechanisms of action of current preservatives can inform the development of novel food preservatives to ensure their efficacy. Here, we used TraDIS-*Xpress*, a large-scale, genome-wide unbiased screen to determine the mechanisms of action of common food preservatives by determining the genes that affect preservative susceptibility in *Salmonella enterica* serovar Typhimurium. We identified genes associated with central metabolism and oxidative stress responses that were important for all four preservatives. Formate dehydrogenase activity and synthesis was crucial for survival in the presence of both sodium chloride and potassium chloride. We found some preservative-specific effects on pathogen susceptibility, for example, LPS synthesis which improved survival upon exposure to sodium nitrite but harmed survival when exposed to sodium chloride or potassium chloride. This research expands our understanding of how some current preservatives act and can inform the effective use of preservatives in current and emerging food products to ensure high standards of food safety.

## Data Availability

Sequence data supporting the analysis in this study have been deposited in ArrayExpress (EMBL-EBI) under the accession number E-MTAB-14856. The authors confirm that all supporting data, code and protocols have been provided within the article or through supplementary data files.

## Introduction

Preservatives are routinely added to food to prevent the growth of pathogens and spoilage bacteria, extending the shelf life of food and ensuring high standards of food safety. Preservatives are thought to act through a combination of reducing water availability, imposing osmotic stress and lowering pH [1,4], but despite their regular use in multiple different products, the genetic basis for their mechanisms of action is often not fully characterized. Whilst currently used compounds have a track record of efficacy, there are growing health concerns about preservatives high in salt including sodium chloride (NaCl), potassium chloride (KCl) and sodium lactate (SL) [5]. In 2015, meat processed by salting, curing [with sodium nitrite (SN) or nitrite salts], fermenting or smoking was classified as carcinogenic to humans by the International Agency for Research on Cancer [6]. Understanding the mechanisms of action of commonly used preservatives and the mechanisms of susceptibility of common food pathogens is therefore necessary to inform the development of novel food preservatives to ensure an equal or greater efficacy at reducing pathogen load and food spoilage.

Our previous work used experimental evolution to determine how planktonic cultures and biofilms of *Salmonella enterica* serovar Typhimurium respond in the presence of preservatives [7]. This found that each preservative selected for changes in biofilm formation and cross-preservative susceptibility in a unique way and preservative exposure did not select for changes in antibiotic susceptibility in planktonic culture or in biofilms. Whole-genome sequencing of populations continuously exposed to these preservatives found that mutations were selected in genes with roles in carbohydrate metabolism, transmembrane transport, stress responses, lipopolysaccharide (LPS) synthesis and flagella synthesis, with no single mutation shared across all four preservative-exposed populations. This gave some insight into the mechanisms of action of each of these preservatives but was not sufficient to determine the full suite of genes that affect preservative susceptibility.

Large-scale transposon insertion sequencing screens are commonly used to identify genes and pathways that affect susceptibility to antimicrobial compounds [8]. These screens expose large pools of unique mutants to compounds that select for mutants with a fitness advantage and select against mutants where a transposon insertion has weakened their chances of survival. Mutant frequency is compared in exposed and unexposed conditions via high-throughput sequencing to determine the genes and pathways involved in susceptibility. TraDIS-*Xpress* is a recent development of this method which incorporates an outward-transcribing promoter into the transposon, creating libraries where every gene in the genome can be overexpressed, as well as inactivated [9]. This facilitates investigation into how gene expression, as well as gene interruption, affects susceptibility and also allows investigation of essential genes, which cannot be inactivated and therefore assayed by conventional approaches.

Here, we used TraDIS-*Xpress* to determine the genes involved in the survival of *S*. Typhimurium in the presence of four common food preservatives: NaCl, KCl, SL and SN. We found that genes involved in LPS synthesis, oxidative metabolism and formate dehydrogenase synthesis had the most significant effect on preservative susceptibility, and the overall pattern of pathways involved suggested that oxidative stress was one of the key mechanisms for preservative-mediated pathogen control. Understanding the mechanisms through which preservative susceptibility is affected will help inform the development of new preservatives and preservative combinations to ensure food safety.

## Methods

### Bacterial strains and plasmids

All strains and plasmids used in this study are listed below (Table 1).

**Table 1.** Bacterial strains and plasmids used in this work

Name	Study
*S. enterica* subsp. *enterica* serovar Typhimurium TraDIS-*Xpress* Tn5 mutant library	[10]
*S. enterica* subsp. *enterica* serovar Typhimurium strain 14028S (WT)	ATCC 14028
Δ*crp*	[7]
Δ*rpoS*	[7]
Δ*rfaG*	[10]
Δ*rfbF*	This study
Δ*rfbU*	This study
Δ*sucA*	This study
Δ*fdoG*	This study
Δ*fdhD*	This study
Δ*fdhE*	This study
Δ*barA*	This study
Δ*fdnG*	This study
Δ*fdoG* Δ*fdnG*	This study

### TraDIS-*Xpress* exposure conditions

The *S. enterica* serovar Typhimurium strain 14028*S* transposon mutant library used in these experiments has been described by Holden *et al.* [10]. The *lacIZ* operon was incorporated into this strain to facilitate control of the transposon-located outward-transcribing *tac* promoter [10]. Approximately 10^7^ c.f.u. ml^−1^ of this library was added to 5 ml lysogeny broth (LB) made without salt in universal tubes and treated with subinhibitory concentrations of each preservative: this equated to 5% NaCl, 10% KCl, 5% SL or 0.125% SN, alongside an untreated control. Transcription from the transposon-located promoter was induced in each condition with 1 mM IPTG, and all conditions used two independent replicates. Cultures were incubated at 37 °C shaking at 200 r.p.m. for 24 h before being centrifuged at 3,000 ***g*** for 10 min to pellet the cells for DNA extraction.

### TraDIS-*Xpress* sequencing library preparation and analysis

DNA was extracted following the protocol described by Trampari *et al.* [11], quantified by Qubit HS dsDNA kit and normalized to 11.1 ng µl^−1^. DNA was tagmented using a MuSeek DNA fragment library preparation kit (Thermo Fisher) and purified using AMPure XP beads (Beckman Coulter). DNA containing the transposon was amplified using primers customized for the tagmented ends and biotinylated primers specific to the transposon. Following another purification step, biotinylated PCR products were incubated for 4 h with streptavidin beads (Dynabeads^®^ kilobaseBINDER^™^, Invitrogen) to capture only fragments containing the transposon. A second PCR step using this captured DNA used customized barcoded Illumina sequencing primers specific to the tagmented ends and to the transposon. Beads were removed from the reaction with a magnet, and DNA was purified and size-selected using AMPure beads. Fragment length was quantified using a TapeStation (Agilent) and sequenced on a NextSeq 500 using the NextSeq 500/550 High Output Kit v2.5 with 75 cycles.

Fastq files were aligned to the *S*. Typhimurium 14028*S* reference genome (CP001363, modified to include chromosomally integrated *lacIZ*) using BioTraDIS (version 1.4.3) [12]. Significant differences (*P*<0.05, after correction for false discovery) in insertion frequencies between the unstressed controls and treated conditions were found using the tradis_comparison.R command (part of the BioTraDIS toolkit) and AlbaTraDIS (version 1.0.1) [13] using default parameters. For all candidate loci, plot files generated by BioTraDIS were examined manually in Artemis (version 17.0.1) [14] to verify the results from the two analysis approaches, as well as to identify regions where inserts were under differential selection but did not fall within coding regions of the genome.

### Construction of gene deletion and overexpression mutants

A selection of genes highlighted by the TraDIS-*Xpress* data was chosen for phenotypic validation. Gene deletion mutants were constructed following the gene doctoring approach by Lee *et al.* [15], using vectors created by Golden Gate assembly as outlined by Thomson *et al.* [16]. Primers for the construction of these vectors are listed in Table S2, available in the online Supplementary Material. Plasmid assembly was confirmed by Sanger sequencing, and deletion mutants were verified by whole-genome sequencing.

### Preservative susceptibility testing via growth analysis

Mutants constructed in *S*. Typhimurium were grown in the presence of each preservative and compared to the WT to investigate how expression of target genes affected preservative susceptibility. Liquid cultures of each mutant were diluted to ~10^5^ c.f.u. ml^−1^ and grown for 18 h in a 96-well plate in the presence of each preservative (5% NaCl, 7.5% KCl, 10% SL or 0.25% SN) alongside an untreated control of LB broth made without salt. The subinhibitory concentrations of each preservative used in this assay were different to those used in the TraDIS-*Xpress* exposure experiments because inhibitory concentrations were affected by different bacterial inoculum densities used in each assay. The OD (absorbance at 600 nm) of each culture was measured every 20 min for 18 h, and the area under each growth curve was calculated using the ‘AUC’ function from the R package ‘DescTools’. Two biological replicates each consisted of three technical replicates. Significant differences between the WT and each mutant exposed to each preservative were determined using a two-way ANOVA with Tukey post hoc analysis.

### Mutant abundance assays

Competition assays between *S*. Typhimurium mutants and the WT identify where intraspecies competition affects preservative susceptibility, and this approach mirrors the pool of mutants used in TraDIS-*Xpress* experiments. To differentiate between mutant and WT populations, the *lacZ* reporter gene was incorporated into the WT chromosome to facilitate blue–white screening on agar supplemented with 1 mM IPTG and 40 µg ml^−1^ X-gal. This marked WT strain has previously been shown to be statistically equivalent to the WT in competition and growth assays [17], and we confirmed no statistical difference in abundance over time when exposed to each preservative stress (Fig. S3). Liquid cultures of *S*. Typhimurium mutants and the marked WT were normalized to an OD of 0.1 and added to a 96-well plate in equal volumes. Cultures were grown for 24 h in the presence of each preservative (5% NaCl, 7.5% KCl, 10% SL or 0.25% SN) alongside an untreated control of LB broth made without salt. At the point of inoculation and after 24 h of growth, 20 µl of culture was taken from each population pool, and serial dilutions were spotted on LB agar made without salt supplemented with 1 mM IPTG and 40 µg ml^−1^ X-gal. Blue and white colonies were counted on inoculation and following 24 h of growth, and the change in mutant abundance over time was calculated. The same assay was repeated under anaerobic conditions with preconditioned anaerobic media in an anaerobic cabinet to determine whether oxygen affected the fitness of target genes when exposed to each preservative. All mutant abundance assays consisted of two biological and three technical replicates.

## Results

### TraDIS-*Xpress* identifies common and preservative-specific mechanisms of susceptibility

**Fig. 1.**
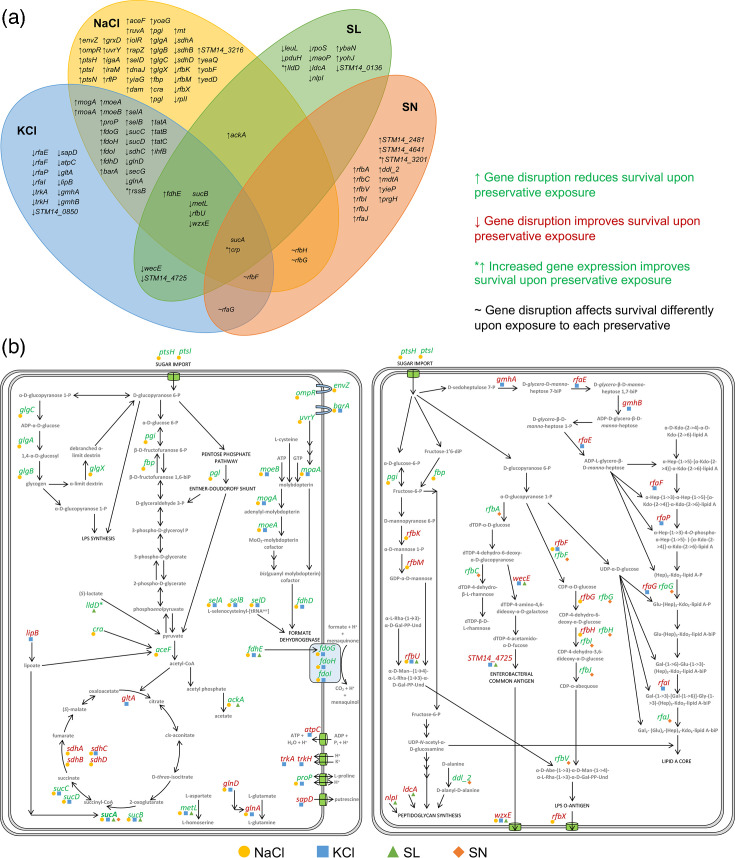
(a) Genes in *S*. Typhimurium that affect susceptibility to NaCl, KCl, SL and SN. (**b**) Genes mapped to metabolic models showing respiration (left) and membrane polysaccharide synthesis (right).

An *S*. Typhimurium transposon mutant library of ~500,000 unique mutants [10] was cultured for 24 h in the presence of 5% NaCl, 10% KCl, 5% SL or 0.125% SN alongside an untreated control. These concentrations were chosen to reduce the growth of *S*. Typhimurium by ~50% (Fig. S1). Total DNA was recovered from all experiments, and mutant abundance was quantified by sequencing. Differences in insertion frequencies per gene between replicates were low, indicating high experimental reproducibility (Fig. S2). Transposon insertion frequency and loci from these conditions were compared to an untreated control, which identified 73 genes that affected survival of *S*. Typhimurium in the presence of NaCl, 47 genes with KCl, 21 genes with SL and 19 genes with SN (Fig. 1a, Table S1). These genes were mapped onto cellular pathways to identify mechanisms of preservative action (Fig. 1b). This found common genes among all four preservative exposures with roles in central metabolism and LPS biosynthesis.

### LPS synthesis has a preservative-specific effect on susceptibility

**Fig. 2.**
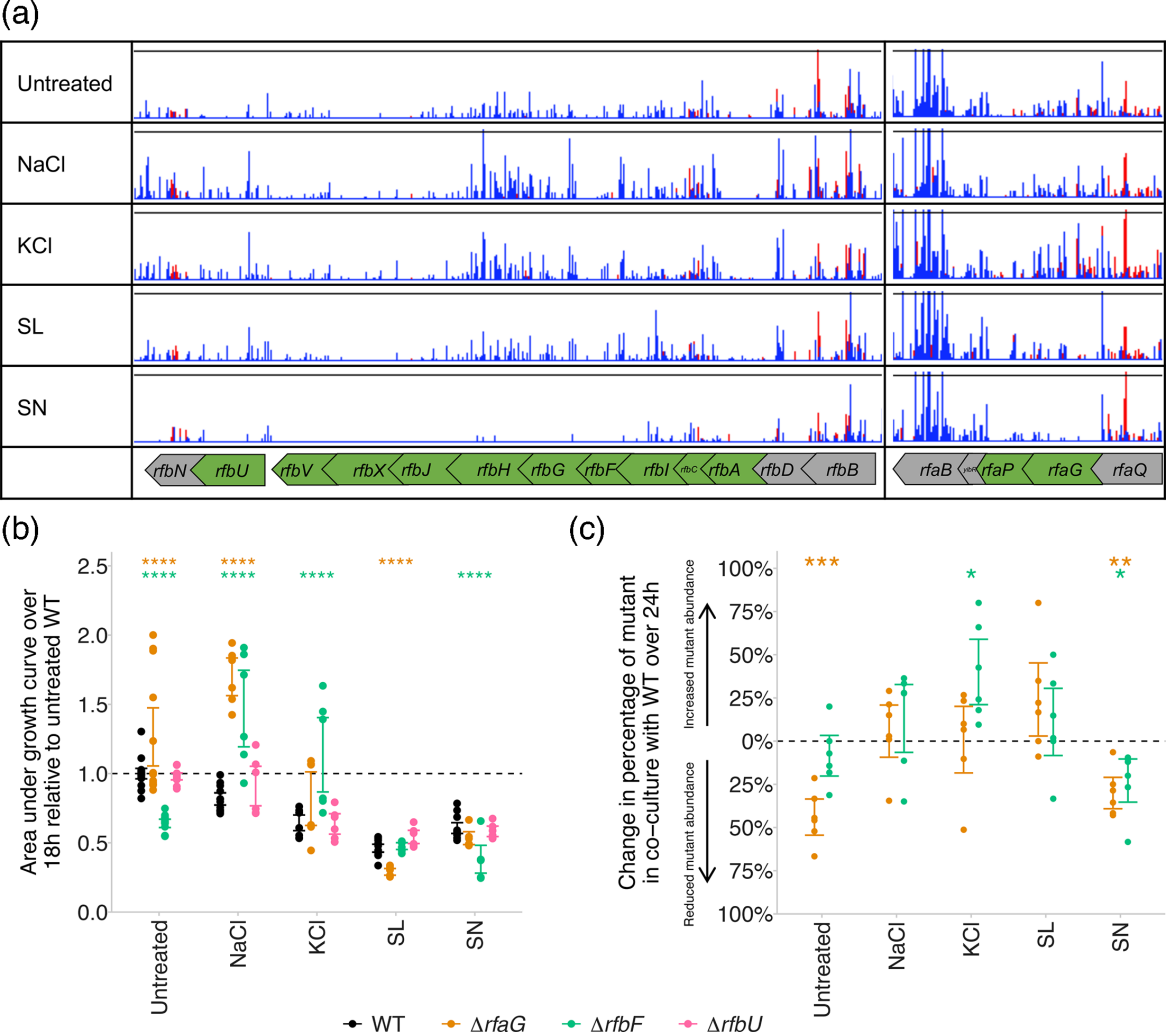
(a) Transposon insertion sites and frequencies in and around genes involved in LPS biosynthesis when treated with NaCl, KCl, SL and SN, relative to an untreated control. Genes highlighted in green were found by TraDIS-*Xpress* to affect growth in the presence of at least one of the preservatives tested (genes in grey are included for context). Red peaks show insertions on the forward strand, and blue peaks show insertions on the reverse strand. Y-axes have been normalized for each locus to show relative differences in insert abundance between conditions. (**b**) Growth of *S*. Typhimurium deletion mutants relative to the untreated WT in the presence of each preservative. Asterisks show significant differences between the WT and mutant exposed to each preservative (two-way ANOVA with Tukey post hoc analysis). (**c**) Change in the percentage of mutants in co-culture with WT *S*. Typhimurium over 24 h. Asterisks show changes in mutant abundance over 24 h (paired *t*-test). For both plots, points show a minimum of two biological and three technical replicates, and error bars denote 95% CIs (*P<0.05, **P<0.01, ***P<0.001 and ****P<0.0001).

Transposon insertions inactivating LPS biosynthesis genes improved survival in the presence of NaCl (*rfbK*, *rfbM*, *rfbU*, *rfbX*, *rfbH*, *rfbG* and *rfbF*), KCl (*rfbU*, *rfbF*, *rfaG*, *rfaE*, *rfaF*, *rfaP* and *rfaI*) and SL (*rfbU*) but, in contrast, reduced survival in the presence of SN (*rfbV*, *rfbJ*, *rfbH*, *rfbG*, *rfbF*, *rfbI*, *rfbC*, *rfbA*, *rfaG* and *rfaJ*) (Fig. 2a). To validate, gene deletion mutants were constructed in three of these genes as representatives of different stages of the LPS biosynthetic pathway: *rfaG*, involved in LPS core biosynthesis, and *rfbF* and *rfbU*, involved in LPS O-antigen biosynthesis. These gene deletion mutants were then grown in the presence of subinhibitory concentrations of each preservative. Supporting our TraDIS-*Xpress* data, mutants lacking *rfbF* had significantly improved growth in the presence of NaCl and KCl and reduced growth in the presence of SN, relative to the WT (Fig. 2b). Mutants lacking *rfaG* had improved growth relative to the WT when exposed to NaCl but reduced growth when exposed to SL (Fig. 2b). Deletion of *rfbU* did not affect susceptibility to any of the four preservatives (Fig. 2b).

TraDIS-*Xpress* experiments are performed in mutant pools, not in isogenic mutant cultures. To validate the findings from these exposure experiments, we co-cultured these gene deletion mutants with WT *S*. Typhimurium to determine the effect of intraspecies competition on mutant abundance in the population upon preservative exposure. We previously engineered a strain of WT *S*. Typhimurium with the *lacZ* reporter gene integrated on the chromosome to facilitate blue–white screening of the WT and mutant populations [17], which we used here. Percentages of each population in the co-culture were determined at inoculation and after 24 h, when the percentage change in mutant abundance was calculated. Supporting the TraDIS-*Xpress* findings, mutants lacking *rfaG* had a significantly higher competitive fitness when exposed to NaCl and KCl, and mutants lacking *rfbF* were significantly more fit when exposed to KCl, relative to the untreated control (Fig. 2c). The effect of LPS on susceptibility to SL was dependent on intraspecies competition: exposure to SL reduced the growth of an *rfaG* deletion mutant in isogenic culture (Fig. 2b) but improved mutant abundance when in co-culture with the WT (Fig. 2c).

We also found that disruption of genes involved in the synthesis of peptidoglycan (*ldcA* and *nlpI*) and enterobacterial common antigen (*wecE* and *STM14_4725*) reduced susceptibility to SL, demonstrating the importance of the bacterial envelope as a whole in preservative susceptibility. Overall, this shows the conditional importance of LPS in preservative susceptibility and highlights how differences in cell envelope biosynthesis affect susceptibility in a preservative-specific manner.

### All four preservatives affect central metabolism and oxidative stress responses

**Fig. 3.**
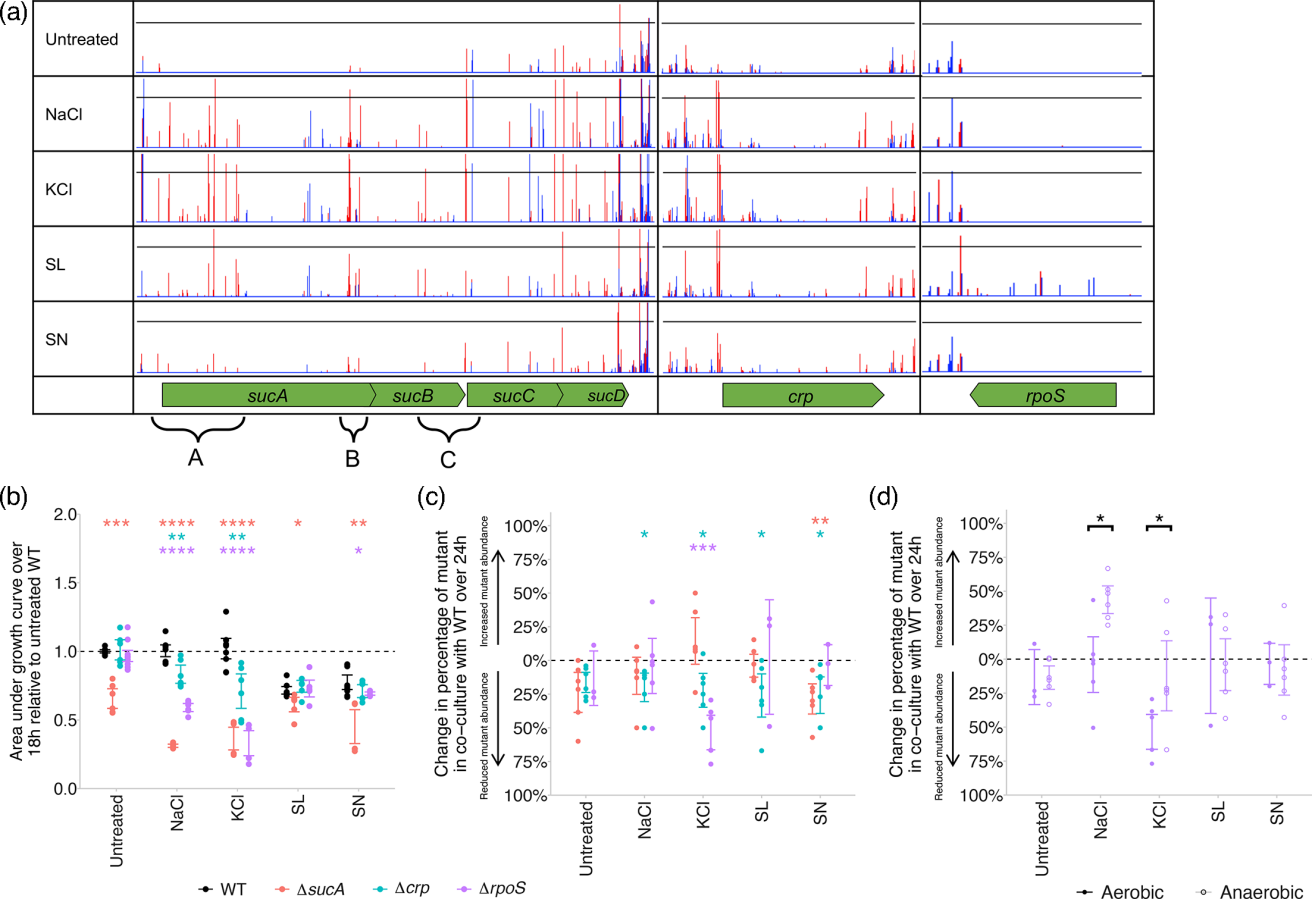
(a) Transposon insertion sites and frequencies in and around *sucA*, *crp* and *rpoS* when treated with NaCl, KCl, SL and SN, relative to an untreated control. Red peaks show insertions on the forward strand, and blue peaks show insertions on the reverse strand. Y-axes have been normalized for each locus to show relative differences in insert abundance between conditions. Regions highlighted by ‘A’, ‘B’ and ‘C’ show where insertions indicate that overexpression of downstream genes could improve survival upon exposure to preservatives. (**b**) Growth of *S*. Typhimurium deletion mutants relative to the untreated WT in the presence of each preservative. Asterisks show significant differences between the WT and gene deletion mutant exposed to each preservative (two-way ANOVA with Tukey post hoc analysis). (**c**) Change in the percentage of mutants in co-culture with WT *S*. Typhimurium over 24 h of growth when exposed to preservative stress. Asterisks show significant changes in mutant abundance over 24 h (paired t-test). (**d**) Change in the percentage of Δ*rpoS* deletion mutants in co-culture with WT *S*. Typhimurium over 24 h of growth under aerobic and anaerobic conditions. Asterisks show significant differences in mutant abundance between aerobic and anaerobic conditions (Welch’s t-test). For all scatter plots, points show a minimum of two biological and three technical replicates, and error bars denote 95% CIs (*P<0.05, **P<0.01, ***P<0.001 and ****P<0.0001).

The TraDIS-*Xpress* screen showed a significant log_2_-fold increase in insertions in *sucA* exposed to each of the four preservatives relative to the unexposed control, indicating that interruption of *sucA* improved survival upon exposure to each of the preservatives tested: there were 5.5 log-fold more insertions when exposed to NaCl, 6.3 log-fold more when exposed to KCl, 6.0 log-fold more when exposed to SL and 2.9 log-fold more when exposed to SN (Fig. 3a). SucA and SucB are two subunits that make up 2-oxoglutarate dehydrogenase, a key component of the TCA cycle supporting central metabolism [18], and there were also more transposon insertions in *sucB* upon exposure to NaCl (5.1 log_2_-fold), KCl (6.0 log_2_-fold) and SL (5.7 log_2_-fold) relative to the unexposed control. Visual examination of the insertion sites mapped to the genome found that the insertions in these conditions were mainly in three distinct areas of the *sucABCD* operon (labelled ‘A’, ‘B’ and ‘C’ in Fig. 3a). TraDIS-*Xpress* differs from conventional transposon sequencing techniques via the use of outward-transcribing promoters inside the transposon, allowing investigation into how increased expression of genes downstream of the transposon can affect survival. Promoters within transposons inserted in region ‘A’ may be driving expression of an essential region of *sucA* immediately downstream of region ‘A’, and insertions in regions ‘B’ and ‘C’ may be driving expression of *sucB* and *sucC*, respectively, which would indicate that increased expression of these genes improved survival upon preservative exposure. A deletion mutant in *sucA* was constructed and grown in the presence of each of the four preservatives, which found a significant reduction in growth upon exposure to all preservatives relative to the WT (Fig. 3b). A *sucA* deletion mutant was also exposed to each preservative in co-culture with the WT, which found a significant reduction in the mutant when exposed to SN (Fig. 3c). This is all consistent with our hypothesis that expression of *sucA* improves survival in the presence of preservatives.

Cyclic AMP receptor protein, encoded by *crp*, was also predicted to improve survival in each of the four preservative-stressed conditions. There were more insertions upstream of *crp* in all conditions tested, indicating that mutants with increased expression of *crp* from the transposon-located outward-oriented promoter have improved survival in the presence of preservatives (Fig. 3a). Cyclic AMP receptor protein is a global regulator that represses the expression of genes involved in central metabolism [19]. We created a mutant lacking a functional copy of *crp* (Δ*crp*) and found reduced growth relative to the WT when exposed to NaCl and KCl (Fig. 3b). In co-culture with the WT, we saw that the *crp* deletion mutant was significantly reduced when exposed to each of the four preservatives (Fig. 3c). This supports our findings in the TraDIS-*Xpress* screen, where expression of *crp* is important for survival in the presence of each of the four preservatives.

Sigma factor S, encoded by *rpoS*, is a global stress response regulator [20] with a characterized role in the general stress response [21,22]. Expression of *rpoS* was found by TraDIS-*Xpress* to affect preservative susceptibility, where 4.1 log-fold more insertions mapped to *rpoS* relative to untreated controls when exposed to SL (Fig. 3a). Conversely, we found that an *rpoS* deletion mutant had significantly reduced growth relative to the WT in the presence of NaCl, KCl and SN (Fig. 3b) and was significantly reduced following exposure to KCl in co-culture with the WT (Fig. 3c). The same intraspecies competition assay previously described was repeated in an anaerobic cabinet to infer whether oxygen affected preservative susceptibility in an *rpoS* deletion mutant. We showed that an *rpoS* deletion mutant was significantly more fit in co-culture under anaerobic conditions relative to aerobic conditions when exposed to NaCl and KCl (Fig. 3d). This implies that preservative exposure may induce a switch from aerobic to anaerobic respiration to avoid the production of reactive oxygen species and that mutants lacking *rpoS* have increased fitness in these conditions.

In addition to genes already described and validated, TraDIS-*Xpress* found that the five genes with the largest significant log_2_-fold changes in transposon insertion frequency between SL-exposed conditions and the control were all in genes reported to affect the oxidative stress response and oxidative metabolism: *pduH* (6.8) [23], *sucA* (6.0) and *sucB* (5.7) [24], *rpoS* (4.1) [20] and *ybaN* (−3.7) [25].

**Fig. 4.**
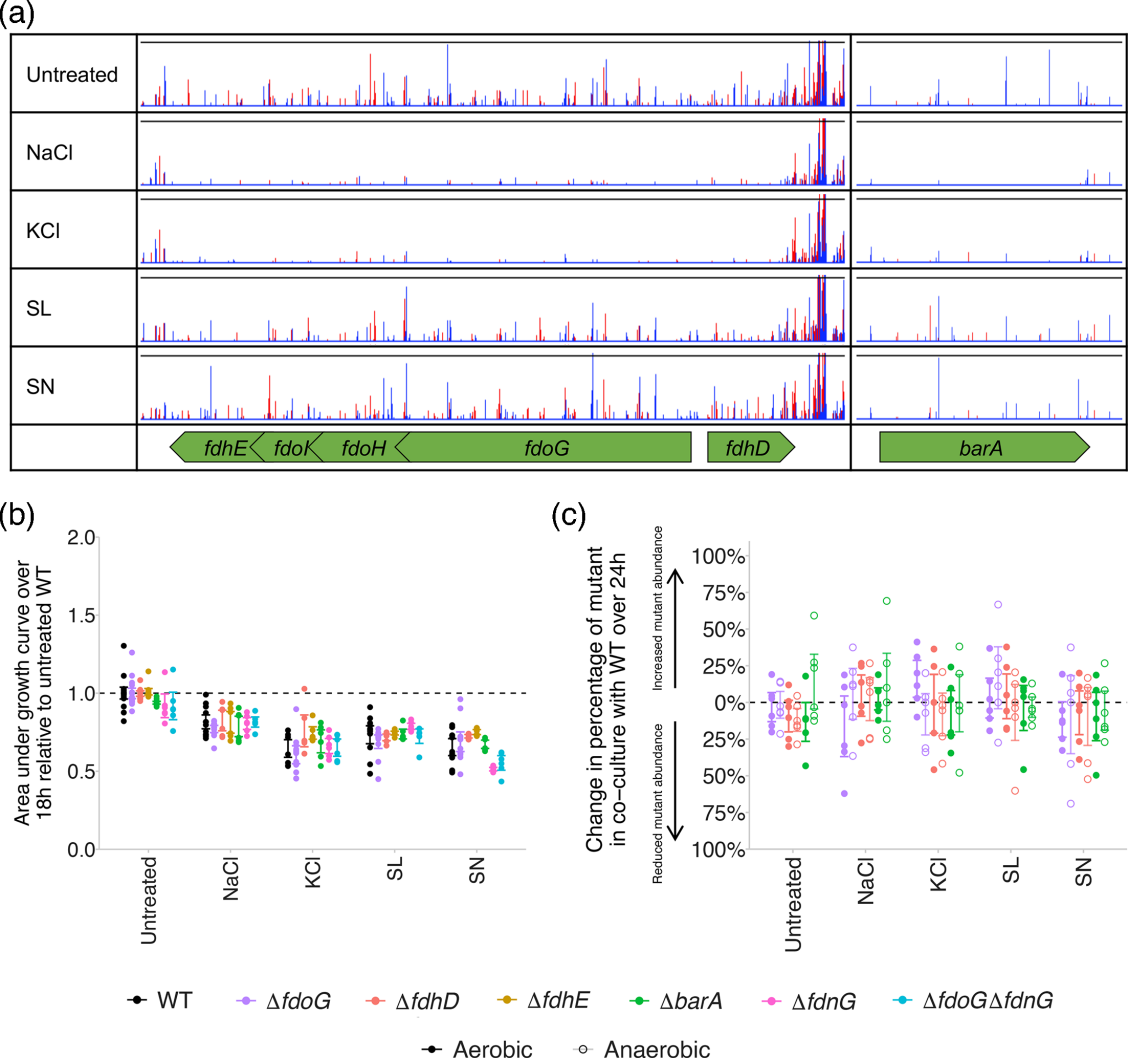
(a) Transposon insertion sites and frequencies in and around genes involved in formate dehydrogenase synthesis and activity when treated with NaCl, KCl, SL and SN, relative to an untreated control. Red peaks show insertions on the forward strand, and blue peaks show insertions on the reverse strand. Y-axes have been normalized for each locus to show relative differences in insert abundance between conditions. (**b**) Growth of *S*. Typhimurium deletion mutants relative to the untreated WT in the presence of each preservative. Asterisks show significant differences between each gene deletion mutant and the WT exposed to each preservative (two-way ANOVA with Tukey post hoc analysis). (**c**) Change in the percentage of deletion mutants in co-culture with WT *S*. Typhimurium over 24 h of growth in aerobic and anaerobic conditions. No significant changes in mutant abundance were found following 24 h of growth (paired t-test) or between aerobic and anaerobic conditions (Welch’s t-test). For both scatter plots, points show a minimum of two biological and three technical replicates, and error bars denote 95% CIs (*P<0.05, **P<0.01, ***P<0.001 and ****P<0.0001).

Formate dehydrogenase synthesis and activity improves survival when exposed to NaCl and KCl. Three genes encoding formate dehydrogenase O (*fdoG*, *fdoH* and *fdoI*) were beneficial to survival in the presence of NaCl and KCl, with up to 3.2 log-fold fewer insertions in these genes seen relative to the untreated control (Fig. 4a). Formate dehydrogenase O is one of three formate dehydrogenase complexes in *S*. Typhimurium and is expressed in aerobic conditions with a potential role in the switch from aerobic to anaerobic respiration [26]. Formate dehydrogenase N is expressed under anaerobic conditions and by increasing periplasmic nitrite concentrations [26,27], and formate dehydrogenase H does not have a respiratory role and is thought to be involved in fermentation [28]. We also saw that genes involved in formate dehydrogenase biogenesis and activity improved survival in the presence of NaCl and KCl, including those with roles in l-selenocysteine-tRNA^sec^ biosynthesis (*selA*, *selB* and *selD*), *bis*(guanyl molybdopterin) cofactor biosynthesis (*moaA*, *moeA*, *moeB*, *mogA* and *fdhD*), formate dehydrogenase activity (*fdhE*) and the two-component sensory system that induces molybdenum transport for formate dehydrogenase activity (*barA* and *uvrY*) [29] (Fig. 1b).

Gene deletion mutants were constructed lacking genes encoding formate dehydrogenase O subunit *fdoG*, formate dehydrogenase activity regulators *fdhE* and *fdhD* and the sensory histidine kinase *barA*. Deletion of these genes resulted in no significant difference in growth in the presence of any of the preservatives tested in monoculture (Fig. 4b) or co-culture with the WT in aerobic (Fig. 4c). Formate dehydrogenase has a characterized role in oxidative stress tolerance in *Escherichia coli* [30,31], so we repeated the co-culture experiments in an anaerobic cabinet to determine how oxygen affected preservative susceptibility in *fdoG*, *fdhD* and *barA* deletion mutants. This found no significant change in mutant abundance under aerobic and anaerobic conditions when exposed to any of the preservatives (Fig. 4c). We hypothesized that this could be due to functional redundancy, and preservative susceptibility could be rescued upon disruption of formate dehydrogenase O through the activity of formate dehydrogenases N. We constructed a mutant lacking the alpha subunits of both formate dehydrogenases O and N (Δ*fdoG* Δ*fdnG*) and looked for changes in preservative susceptibility in this mutant relative to the WT. Again, we found no significant difference in growth in the presence of any of the preservatives tested in the double mutant relative to the WT or the single-gene deletion mutants (Fig. 4b). The role of formate dehydrogenase activity in preservative susceptibility is therefore not clear and may not be obvious in simple growth or competition assays.

## Discussion

This work using a whole-genome screen builds upon our previous work using experimental evolution to select for mutations following continuous exposure of *S*. Typhimurium to four preservatives [7]. There were five genes highlighted by both studies that affect fitness in the presence of preservative stress: *ompR*, *rfbK*, *crp*, *rpoS* and *tatB*. OmpR is a response regulator in the EnvZ/OmpR two-component system with a well-characterized role in responding to changes in osmotic stress and NaCl [32].

As well as a missense mutation in *rfbK* (involved in LPS O-antigen synthesis), our previous work also found a missense mutation in *lpxO* involved in lipid IV_A_ synthesis of the LPS core following continuous exposure to NaCl [7]. Together with the TraDIS-*Xpress* screen, these data are consistent in showing that disruption of LPS synthesis improved survival in the presence of NaCl and KCl. We also show that the LPS affects preservative susceptibility in a compound-specific manner, where disruption of LPS synthesis reduced survival upon exposure to SN. The difference in the effect of LPS synthesis on susceptibility to NaCl and SN could be a potential target for preservative synergy, as LPS synthesis is both essential and detrimental for survival in the presence of each preservative. We have previously successfully predicted synergies between antimicrobial compounds [33], and this tool could be used for the development of future preservative mixtures and antimicrobials.

Our screen found that increased expression of *crp* reduced susceptibility to each of the four preservatives, and we saw reduced abundance of a *crp* deletion mutant in co-culture with the WT upon exposure to each preservative. This also supports our previous experimental evolution study, which found that a C19Y missense mutation in *crp* was selected by continuous exposure to NaCl, KCl and SL [7]. The cAMP receptor protein encoded by *crp* is involved in regulating carbon metabolism [19], and changes in expression could affect preservative susceptibility downstream, requiring further investigation in future studies. The oxidative stress response has previously been linked to *crp* [34] through reduced intracellular cAMP levels, which reduce the activity of the cAMP receptor protein (encoded by *crp*) and remove the repression of *rpoS*, allowing expression of sigma factor S and induction of the general stress response [21]. Both the screen here and our previous experimental evolution study [7] found that mutations in *crp* and *rpoS* affected susceptibility to NaCl, KCl and SL, which supports our hypothesis that these preservatives induce an oxidative stress response in *S*. Typhimurium. Many genes involved in oxidative metabolism were predicted to be involved in susceptibility to the preservatives, including *sucA* and *sucB* in the TCA cycle, which are a significant source of reactive oxygen species production [35]. Of the 19 genes found to affect susceptibility to SN, 12 had roles in cell envelope synthesis, and only *crp* and *sucA* could be linked to oxidative metabolism. This suggests that barrier integrity has a much stronger effect on susceptibility, but that SN may also induce an oxidative stress response in *S*. Typhimurium. However, *crp* and *rpoS* also have roles in other stress responses such as to heat and acid stress [20,34] and do not solely indicate an oxidative stress response. This should be further investigated by measuring the production of reactive oxygen species following preservative exposure. Expression of *crp* is regulated by cAMP and glucose availability in the cell, whereas *rpoS* accumulation is regulated by a much wider range of effectors, such as osmolarity, pH, temperature, starvation and DNA damage [36]. The regulons of both affect genes involved in energy metabolism and transmembrane transport [20,37] and overlap to reduce sensitivity to a wide range of stresses, thereby providing a level of cross-protection. There is a complex interplay between *crp* and *rpoS*, where cAMP-CRP has been reported to repress *rpoS* accumulation, but cAMP is also necessary for *rpoS* activity across its full regulon [38]. Overall, this suggests coordinated regulation between *crp* and *rpoS* is necessary for survival in the presence of preservatives.

The screen predicted 15 genes directly involved in the synthesis, regulation and activity of formate dehydrogenase in reduced susceptibility to NaCl and KCl, but attempts to validate this prediction with defined mutants with deletions of these genes found no significant effect on preservative susceptibility. Whilst we were able to validate the predicted roles of various pathways from the initial screen with defined mutants, we did not see a phenotype for some targets (including formate dehydrogenase) in validation. This is not uncommon in TnSeq validation, where the primary experiment is usually more sensitive than a follow-up, as signals are based on dozens of mutants in competition within a massive pool rather than individual deletion mutants competing against a WT 1–1. Although we could not validate the predicted role of formate dehydrogenase, others have shown a role for formate dehydrogenase in oxidative stress, where formate is used as a reducing factor to alleviate the effects of decreased NADH seen under oxidative stress [31]. Both our experimental evolution study and the TraDIS-*Xpress* screen found *tatB* to have an important role in preservative stress, and the Tat translocation system is known to be involved in localization and activity of formate dehydrogenase [39], with a previously described role in oxidative stress [40].

Despite the evidence that NaCl, KCl and SL may induce an oxidative stress response in *S*. Typhimurium, we did not see any mutations or differences in insertion frequency in main regulators of the oxidative stress response such as *oxyR* and *soxS*, which are usually seen to regulate and control the cell’s oxidative stress response. We also see no evidence of the cell responding to damage to DNA or proteins caused by radical oxygen species, the canonical method through which oxidative stress kills the cell [22]. This could be due to functional redundancy, whereby interruption of one gene does not result in a significant change in fitness because another gene is overexpressed to compensate. This could also be because the expression of these genes does not provide a significant fitness benefit in a mixed mutant pool when exposed to preservatives. These are limitations of the TraDIS-*Xpress* approach that could, in the future, be overcome by complementing our work with RNA-seq experiments to link gene expression and fitness profiles.

Our findings support previous studies, suggesting that these preservatives induce genes involved in the oxidative stress response: NaCl has been shown to induce genes involved in the oxidative stress response in *E. coli* [41], and KCl and SL have been shown to induce oxidative stress in *Pseudomonas aeruginosa*, an opportunistic pathogen with a substantial role in food spoilage [42]. SN exposure was reported to cause downregulation of anaerobic respiratory pathways [43] and, when converted into nitric oxide, disrupted key enzymes involved in the TCA cycle [44], also seen in our results. Salt is also widely reported to increase the production of reactive oxygen species in plants [45]. Although the mechanism through which preservatives induce an oxidative stress response is unclear, we hypothesize that the energy demand imposed on the cell when exposed to preservatives is such that there is an increase in aerobic respiration and reactive oxygen species generation. This is consistent with our work and others showing the importance of genes involved in central metabolism when exposed to salts [46]. Our work builds upon these previous studies by using a whole-genome unbiased screen to directly compare different preservatives and quantify the relative effects on susceptibility of multiple pathways at once.

Combining both TraDIS-*Xpress* and our previous work using experimental evolution has identified mechanisms of action and susceptibility to common food preservatives in the foodborne pathogen *S*. Typhimurium. All four preservatives appear to induce an oxidative stress response in the cell, but LPS synthesis could be a potential target for cross-preservative synergy. The mechanisms of action and susceptibility highlighted by this work should inform the development of future preservatives and preservative combinations to ensure food safety and limit the emergence of resistant pathogens.

## Supplementary material

10.1099/mic.0.001609 Uncited Supplementary Material 1.

10.1099/mic.0.001609 Uncited Table S1.

## Abbreviations

KCl: potassium chloride
LB: lysogeny broth
LB: lysogeny broth
LPS: lipopolysaccharide
NaCl: sodium chloride
SL: sodium lactate
SN: sodium nitrite
