# Supplementary material for: Common food preservatives induce an oxidative stress response in Salmonella enterica serovar Typhimurium
Source: Microbiology (Reading). 2025 Sep 15;171(9):001609. doi: 10.1099/mic.0.001609 (PMC12440568; doi:10.1099/mic.0.001609)
Supplement: Uncited Supplementary Material 1. [file mic-171-01609-s001.pdf]

Supplementary information

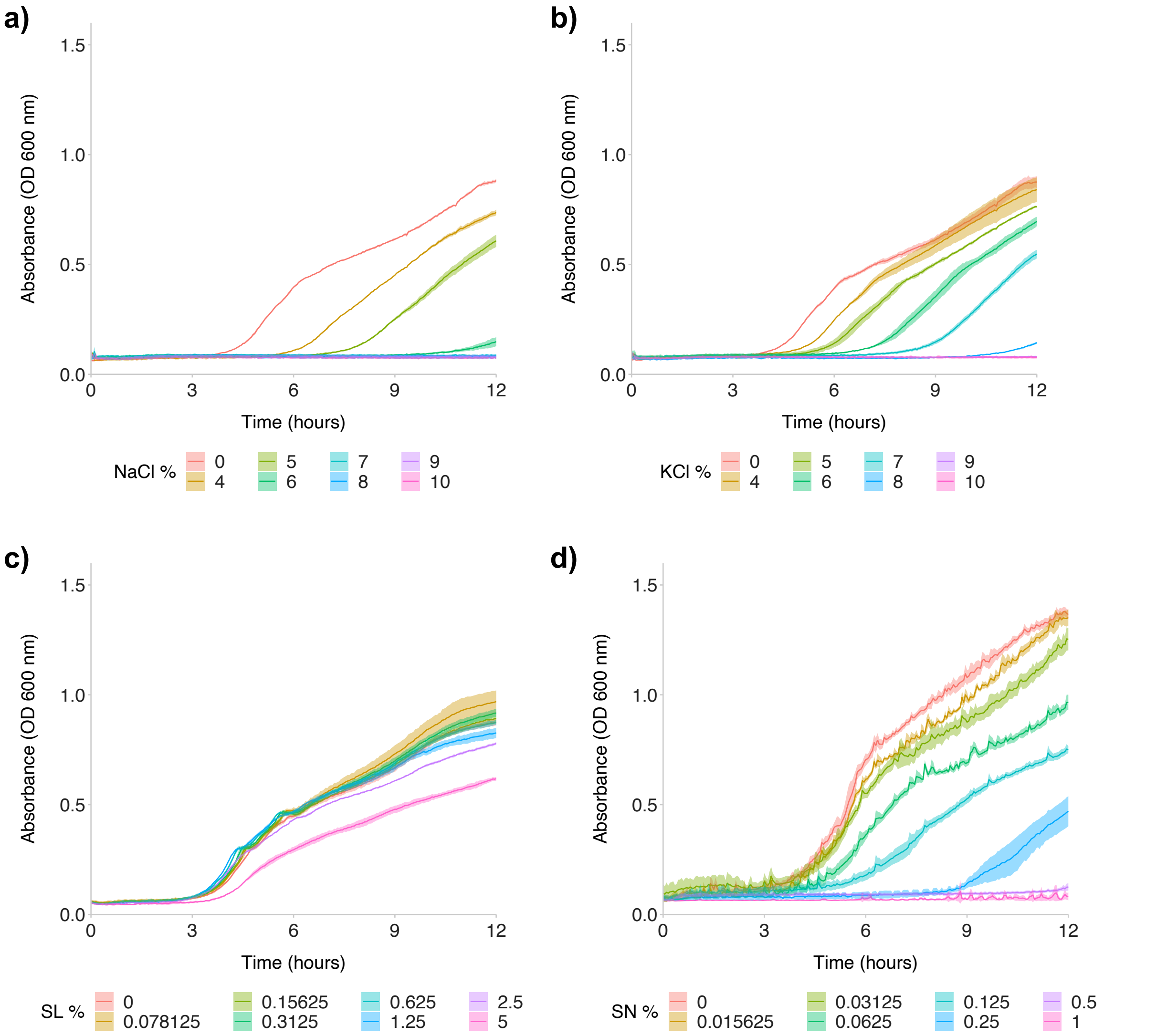

Supplementary figure 1: Growth of S. Typhimurium over 12 hours in the presence of a range of concentrations of **a)** sodium chloride (NaCl), **b)** potassium chloride (KCl), **c)** sodium lactate (SL) and **d)** sodium nitrite (SN). Lines show mean growth over time of three independent replicates and coloured areas show 95% confidence intervals.

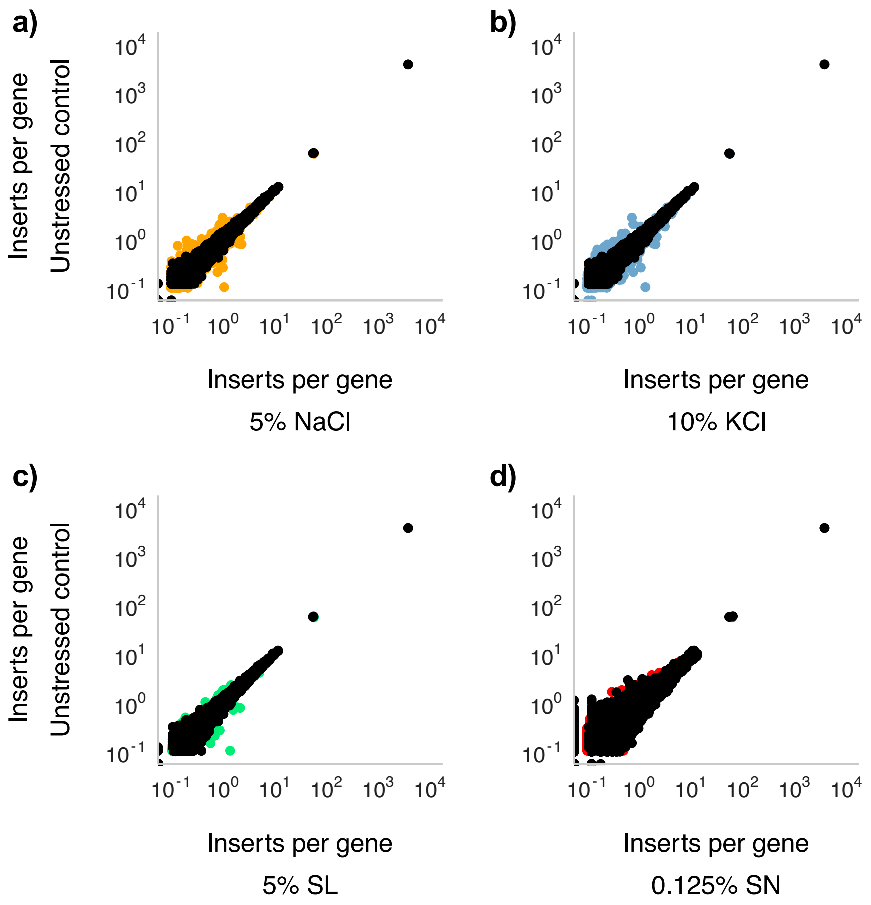

Supplementary figure 2: Transposon insertion frequency per gene. Black points represent variation between the first and second independent replicates, and coloured points show the difference in insertion frequency between the untreated control and conditions treated with **a)** 5% sodium chloride (NaCl), **b)** 10% potassium chloride (KCl), **c)** 5% sodium lactate (SL) and **d)** 0.125% sodium nitrite (SN).

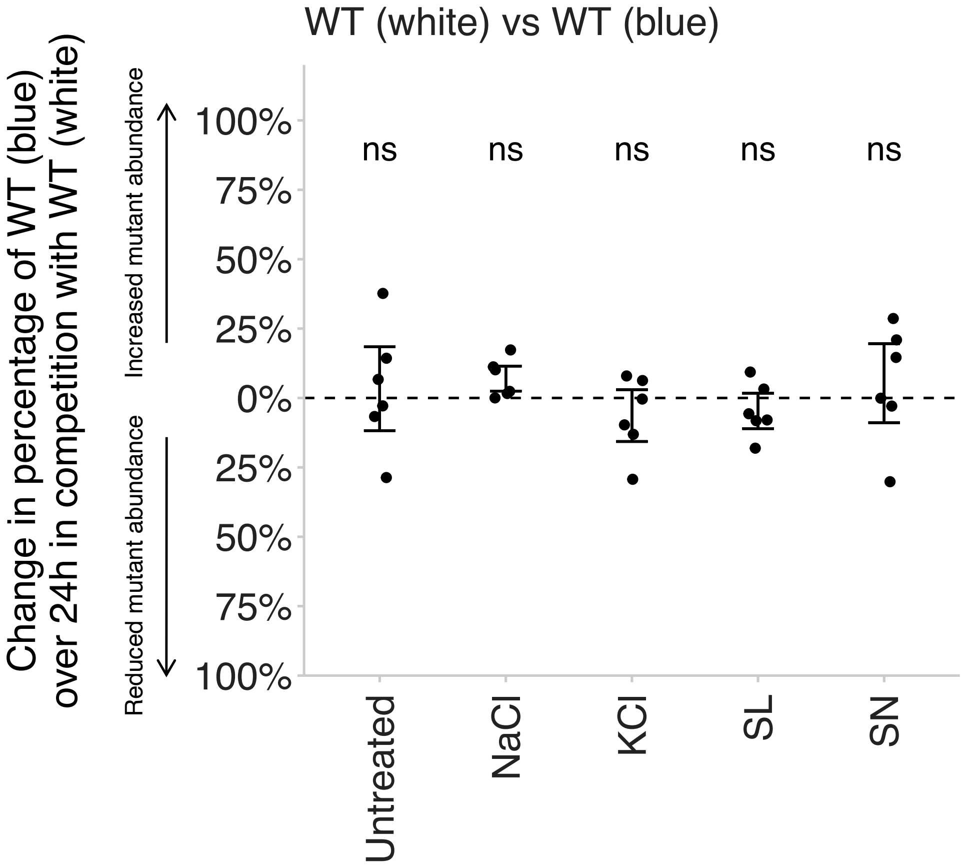

Supplementary figure 3: Change in the percentage of WT S. Typhimurium with *lacIZ* insertion (WT blue) in co-culture with WT S. Typhimurium (WT white) over 24 hours growth. No significant changes in mutant abundance were found following 24-hours growth (Paired t-test) or between the untreated and preservative exposed conditions (Two-way ANOVA with Tukey post-hoc analysis). Points show a minimum of two biological and three technical replicates and error bars denote 95% confidence intervals (ns = not significant).

Supplementary table 2: Primers used to construct plasmids for gene deletion or overexpression.

Name	Description	Primer sequence
rfbF HR1 F	For deletion of *rfbF*	GTCGGGTCTCGCTACACTCACGATTGTCGTAGCACT
rfbF HR1 R	For deletion of *rfbF*	GCTGGTCTCTCTCCTAGATGATTGATAAAAATTTTTGGCAAGGTAAACG
rfbF HR2 F	For deletion of *rfbF*	GTAGGTCTCTCGCTAATTATCCTCAATATTATTAGATGCGGTAAATGCATCAGAA
rfbF HR2 R	For deletion of *rfbF*	GTGGGTCTCATCGTTTTTTTATTCGTGAAAGTGACAGACCTATAATCTTCC
rfbU HR1 F	For deletion of *rfbU*	GGGGGTCTCGCTACAACAATAGATTTTGAACTATAATTAAAATTTCTGG
rfbU HR1 R	For deletion of *rfbU*	GGGGGTCTCTCTCCTATTCCCACATATAATGCAGGGTC
rfbU HR2 F	For deletion of *rfbU*	GGGGGTCTCTCGCTTTCCATACGCATCTTTAAAGCAA
rfbU HR2 R	For deletion of *rfbU*	GGGGGTCTCATCGTGTAATAAAAATATGATCAACACTGTACTTCG
sucA HR1 F	For deletion of *sucA*	GGGGGTCTCGCTACGTCTTATCCGGCCTACAGGCG
sucA HR1 R	For deletion of *sucA*	GGGGGTCTCTCTCCGTTCTGCATCGTGATCCCTTAAGC
sucA HR2 F	For deletion of *sucA*	GGGGGTCTCTCGCTGTCGATTAAATAAAGGATAAATAATGAGTAGCGTAGATATTCTTGT
sucA HR2 R	For deletion of *sucA*	GGGGGTCTCATCGTAGCGGGTGCTTTAGACTCGC
fdoG HR1 F	For deletion of *fdoG*	GGGGGTCTCGCTACATTTATAGACGCGGTTGTCTTCCGGGTT
fdoG HR1 R	For deletion of *fdoG*	GGGGGTCTCTCTCCAAGGTGTAACGGAGACGACCTATG
fdoG HR2 F	For deletion of *fdoG*	GGGGGTCTCTCGCTGACCTGCATGGTTTGC
fdoG HR2 R	For deletion of *fdoG*	GGGGGTCTCATCGTATCAATCTGCACTTCAAGTCCGTTAC
fdhD HR1 F	For deletion of *fdhD*	GGGGGTCTCGCTACACCAGACCGGCCCC
fdhD HR1 R	For deletion of *fdhD*	GGGGGTCTCTCTCCATTGTTCACTTTCTTTTAACCATATAAGAACACACACAC
fdhD HR2 F	For deletion of *fdhD*	GGGGGTCTCTCGCTGCAGACTAAAATAGTACCCTCTACATTTGCG
fdhD HR2 R	For deletion of *fdhD*	GGGGGTCTCATCGTATCGTAGCTTCCCTGGCTTTATTTCT
fdhE HR1 F	For deletion of *fdhE*	GGGGGTCTCGCTACAGGCAAAGGAAAGCGTAGTGGCGTG
fdhE HR1 R	For deletion of *fdhE*	GGGGGTCTCTCTCCGGGGAATAAGTTCTCTGGTATACCGGC
fdhE HR2 F	For deletion of *fdhE*	GGGGGTCTCTCGCTAATACTCATTCAGTTTCTTTTTCCGTTGTCTTGCGG
fdhE HR2 R	For deletion of *fdhE*	GGGGGTCTCATCGTCGCAACTGGCGCGAATTC
barA HR1 F	For deletion of *barA*	GGGGGTCTCGCTACATTTGCAGCGGTTTATCCGG
barA HR1 R	For deletion of *barA*	GGGGGTCTCTCTCCGTTGGTCATGGAGTTCCGTTATGGG
barA HR2 F	For deletion of *barA*	GGGGGTCTCTCGCTAGGCTGATATTCGCCGGATGG
barA HR2 R	For deletion of *barA*	GGGGGTCTCATCGTGATTGCCCGCGATCTGCATG
fdnG HR1 F	For deletion of *fdnG*	GGGGGTCTCGCTACGTTCGCGTACTGGATGATAGC
fdnG HR1 R	For deletion of *fdnG*	GGGGGTCTCTCTCCAACATCGAGAAGGCGTAAGGG
fdnG HR2 F	For deletion of *fdnG*	GGGGGTCTCTCGCTCATTGCTTTCCTCTTTTTTCCACGGATG
fdnG HR2 R	For deletion of *fdnG*	GGGGGTCTCATCGTATTAACCCAACCATCGTGCTCCA
crp pJMA F	For inserting crp into pJMAt5 downstream of a t5 promoter	GGGGGTCTCGCATGATGGTGCTTGGCAAACCGC
crp pJMA R	For inserting crp into pJMAt5 downstream of a t5 promoter	GGGGGTCTCGAATTTTAACGGGTGCCGTAGACGA
